# The President’s Address

**Published:** 1867-06

**Authors:** 


THE PRESIDENT'S ADDRESS.

Dr. Askew, the President, then delivered his inaugural address, an hour in length, but pertinent and important as it was comprehensive. He first referred to the origin of the Association, and sketched its usefulness in its history for the past eighteen years, showing how it had elevated and rendered more efficient the medical profession in the United States.

The membership included about three thousand practitioners, scattered in every section of the country, through whose influence the power of the Association was disseminated and exerted among the profession at large. The printing of the reports and papers submitted to the Association, in the volume of annual published proceedings, was instrumental in spreading a knowledge of the facts discovered and experimented upon by the more scientific.

He called attention to the fact that never more than 1100 of these volumes had been distributed, and of late years only 500 volumes, whereas they should be in the hands of every intelligent and progressive physician.

He treated briefly of specialties in medicine. He admitted that the science of medicine, in connection with the endlessly varied forms of disease, was too vast to be comprehended fully and practically by any one mind, and therefore devotion to any one or a few branches of the art of curing disease was not to be condemned; but he suggested that, inasmuch as the special practioner was likely to be unfamiliar with and unskilled in regard to the general symptoms or general causes of special diseases, consultations between the special and general practitioners would be conducive of good results. Neither should entirely ignore the other.

In regard to empyricism, the President said the profession was in some degree responsible for its existence. It had formerly frowned upon all dissemination of medical knowledge among the people, leaving them the prey to pretenders. The teaching of physiology, botany, etc., in our schools, was a step in the right direction, which he hoped would be continued in other departments of medical knowledge.

The subject of a better grading and more thorough instruction in medical colleges was urged upon the consideration of the Association.

The practice of using opium as a stimulant, by respectable people, who condemn the use of intoxicating drinks, was adverted to, and its destructiveness of health and life urged as considerations why physicians should exert their influence to check the evil; that influence might be brought to bear upon our Legislatures, to secure enactments against the sale of the drug, except on prescriptions.

The list of Special Committees were called in order, and such reports as were ready were referred to the appropriate Sections for consideration.

